# Purkinje cell development and degeneration in the spastic Han-Wistar rat model of ataxia

**DOI:** 10.1038/s41598-026-58826-y

**Published:** 2026-06-25

**Authors:** Hanna Bellafard, Tanya Pelayo, Michael Aghopoo, Diba Sarhangnejad, Shayn Gaft, George Radionov, Yasmin Hosseini, Luciana Werneck Zuccherato, Carlos Eduardo G. Amorim, Maria Elena de Bellard

**Affiliations:** 1 Department of Biology, California State University, Northridge, CA USA; 2 Center for Evolution and Medicine, School of Human Evolution and Social Change, Arizona State University, Tempe , CA USA

**Keywords:** Purkinje neuron, Motor control, Hereditary ataxia, Rare disease, Whole Genome Sequencing, Diseases, Genetics, Neurology, Neuroscience

## Abstract

Hereditary ataxia is a neurodegenerative disorder notable for its early onset, with symptoms appearing in patients as young as two years old. Although affected individuals exhibit severe motor deficits and early mortality rates, the timeline of Purkinje cell loss remains unclear. To address this gap, we used the spastic Han-Wistar rat model, which harbors an unknown homozygous recessive variant that causes Purkinje cell loss. Here, we determined the onset and temporal progression of Purkinje neuronal loss in the spastic Han-Wistar model. To achieve this, we employed immunohistochemistry, Hematoxylin and Eosin histology, and neuronal density quantification. Behavioral testing demonstrated early-onset, progressive motor impairment in mutant rats, with motor deficits emerging around P45, following Purkinje cell loss detectable as early as P15. Additionally, guided by pedigree analysis indicating autosomal recessive inheritance for this ataxia, we performed whole-genome shotgun sequencing of a parent-offspring trio to identify amino acid-changing mutations consistent with this pattern. Sanger sequencing excluded Sbf2 as a causal variant, and whole-genome sequencing identified 273 candidate genes consistent with autosomal recessive inheritance. Together, our findings provide new insights into the onset and genetic complexity of ataxia, refining the value of the spastic Han-Wistar rat as a model for investigating mechanisms underlying hereditary ataxia.

**Supplementary Information:**

The online version contains supplementary material available at https://doi.org/10.1038/s41598-026-58826-y.

## Introduction

Neurodegenerative diseases represent one of the most pressing challenges in modern medicine, affecting millions of individuals worldwide and imposing substantial burdens on healthcare systems and families^1^. Among these disorders, hereditary ataxias constitute a clinically and genetically heterogeneous group of conditions characterized by progressive cerebellar dysfunction, resulting in impaired coordination, balance, and motor control^2^. These disorders exhibit substantial phenotypic diversity, with onset ranging from early childhood to late adulthood and inheritance patterns spanning autosomal dominant, autosomal recessive, and X-linked transmission^3^.

The human cerebellum, though comprising only 10% of total brain volume, contains more neurons than the rest of the brain combined and serves as a critical hub for motor coordination and cognitive processing^4,5^. Purkinje cells function as the sole output neurons, integrating vast amounts of sensory information through their intricate dendritic arbors and providing inhibitory signals to the deep cerebellar nuclei^6,7^. These neurons are highly specialized yet particularly vulnerable to genetic mutations and environmental insults, with their degeneration serving as a hallmark of cerebellar ataxias^8,9^. The vulnerability of Purkinje cells has further been documented in other neurodegenerative conditions, including neuronal ceroid lipofuscinosis, where their loss contributes to early motor impairment and disease progression^10^.

While the selective vulnerability of Purkinje cells in ataxia is well recognized, the temporal dynamics of their degeneration in relation to symptom onset remain unclear, which limits opportunities for early intervention strategies^11^. Furthermore, while numerous genetic loci have been associated with hereditary ataxias, approximately 75% of patients remain without a molecular diagnosis, highlighting the incomplete understanding of the genetic architecture underlying these disorders^12,13^.

Animal models have been invaluable for investigating the mechanisms of cerebellar degeneration and testing potential therapeutic interventions^14–16^. The spastic Han-Wistar (sHW) rat, first described in 1976, is a mutant strain that exhibits hyperactivity, spastic paresis, forelimb tremor, and hind limb rigidity, all of which progress over time. This strain represents a well-established model of hereditary ataxia, characterized by progressive motor dysfunction, cerebellar degeneration, and early mortality by approximately postnatal day 60 (P60)^17^.

Previous studies have documented significant Purkinje cell loss and structural abnormalities in the cerebellar cortex; yet, the genetic basis of this mutation remains unknown, and the precise timeline of neurodegeneration has not been systematically characterized^18,19^. Moreover, the developmental progression, reginal distribution, and behavioral phenotype of this model across the full P15–P60 lifespan have not been fully examined. Here, we sought to provide a detailed temporal map of Purkinje cell loss in the sHW rat model, demonstrating that degeneration begins earlier than previously reported P30 and precedes the onset of motor symptoms^20^. In addition, we present a comprehensive temporal investigation of Purkinje cell degeneration in the sHW rat model, combining behavioral assessments and histological analyses to address two fundamental questions: (1) What is the temporal progression of Purkinje cell loss relative to the onset of motor symptoms? and (2) What is the progression of motor impairments in mutant rats as measured by rotarod, open-field, and weight analysis? Through Immunohistochemistry (IHC), Hematoxylin and Eosin (H&E) staining, and quantitative neuronal density measurements, we demonstrate that Purkinje cell degeneration begins as early as P15, preceding the emergence of overt motor deficits. Additionally, we employed whole-genome sequencing and Sanger validation to identify candidate genes potentially responsible for the disorder. Our findings provide new insights into the pathological timeline of cerebellar degeneration and contribute to the growing understanding of genetic factors underlying hereditary ataxias.

## Results

This study investigated the progression and timeline of Purkinje cell degeneration in the cerebellum of sHW mutant rats and examine the genetic basis underlying this trait. To address these goals, we employed three experimental approaches: (i) behavioral assays including Open Field, Rotarod; (ii) histological analyses using H&E staining and IHC; and (iii) whole-genome shotgun sequencing and Sanger sequencing to identify candidate genes underlying the trait.

### Early-onset motor and systemic decline in ataxic rats

**Fig. 1:**
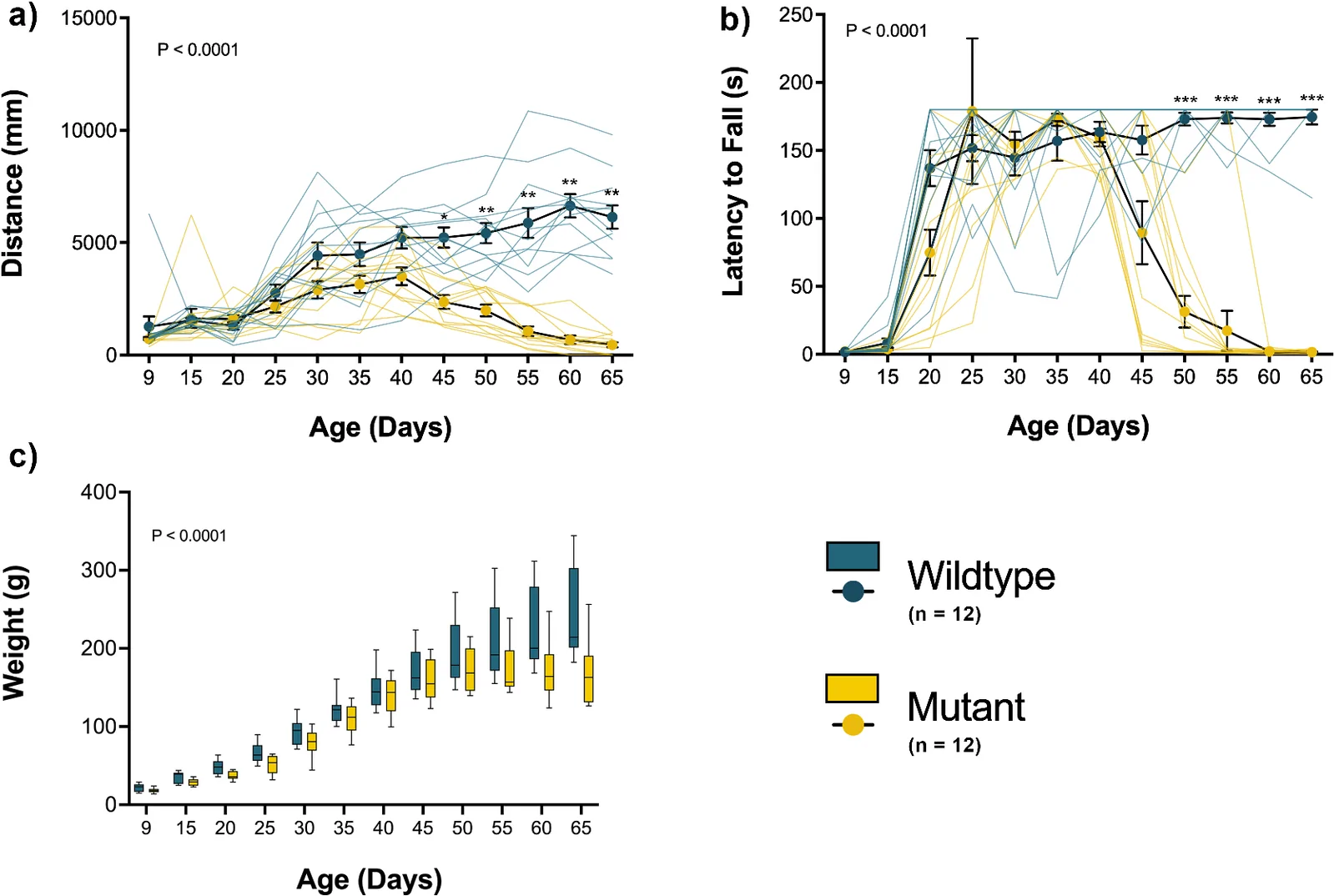
Behavioral assessment of spastic Han-Wistar rats. Behavioral assessments were performed on wild-type and mutant rats (*n* = 12 per group) every 5 days from P9 to P65. Points represent group means ± SEM with individual animal trajectories shown in the background. (**a**) Locomotor activity measured as total distance traveled in the open-field test (120 s duration). (**b**) Motor coordination assessed by latency to fall in the rotarod test (180 s duration). (**c**) Body weight measurements over the same period are used to monitor changes in body mass. Statistical analysis was performed in all behavioral tests using repeated-measures ANOVA. Error bars represent the standard error of the mean.

Motor dysfunction is a defining feature of the spastic Han-Wistar phenotype. To assess how motor impairments progress across development in mutant rats, we performed a series of behavioral tests on *n* = 12 mutant and *n* = 12 wild-type rats. Locomotor activity, measured by the open-field test, was significantly reduced in mutant rats compared to wild-type controls. The total distance traveled declined progressively with age (Fig. 1a), reflecting reduced mobility. Wild-type rats traveled an average of 4,190 millimeters compared to 1,846 millimeters in spastic mutants, representing a 56% reduction in locomotor activity (two-way repeated measures ANOVA, *p* < 0.0001; mean difference = 2,344 units, 95% CI: 1,616-3,072). Motor coordination, assessed using the rotarod test, also declined over time in mutants (Fig. 1b). Wild-type rats remained on the rotarod for an average of 134.6 s compared to 73.97 s for spastic mutants, representing a 45% reduction in motor coordination performance (two-way repeated measures ANOVA, *p* < 0.0001; mean difference = 60.65 s, 95% CI: 42.24–79.07). Mutant animals exhibited early signs of motor impairment, falling before reaching the traditional target duration of 180 seconds^21^. Deficits became prominent by P45, at which point most mutants were unable to complete the trial, indicating impaired balance and coordination. Body weight measurements of mutant rats and controls were recorded, and gradual pattern of weight loss was observed in mutants, consistent with overall systemic decline across development (Fig. 1c). These findings provide a comprehensive view of the behavioral differences between mutant and control rats, capturing how the phenotype progresses across the full developmental span from P9 to P65.

### Disrupted morphology and reduced cell counts by H&E

Previous studies examined Purkinje cell morphology only at P30 and older; here we investigated whether structural degeneration occurs earlier than previously recognized^20^. To assess the morphology and physical appearance of Purkinje cells in the cerebellum, we utilized the H&E staining on the cerebellum of sHW mutants and wild-type rats at P15, P30, and P60. Cerebellar sections from *n* = 3 mutant and *n* = 3 wild-type rats were analyzed per time point, with fifteen randomly selected sections per animal examined and counts averaged across sections.

**Fig. 2:**
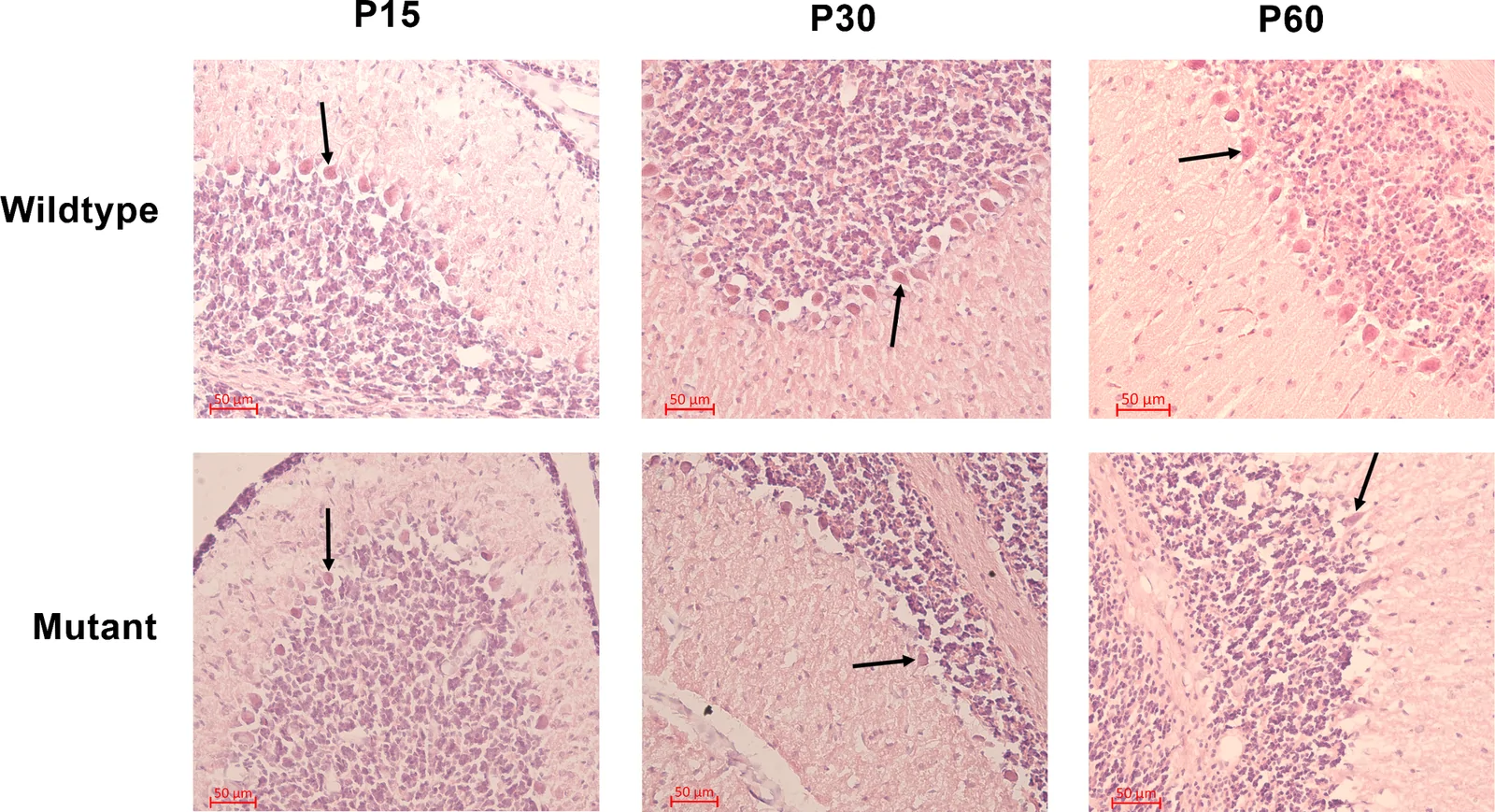
Hematoxylin & Eosin staining of Purkinje cells in the cerebellum of Spastic Han-Wistar rats. Representative H&E-stained sections (40x; scale bar = 50 μm) of wild-type and mutant at P15, P30, and P60. Images are representative of the overall pattern observed throughout the cerebellum across all lobules. Wild-type rats show a single, well aligned Purkinje cell layer, whereas mutants display reduced cell number and disrupted morphology. *n* = 3 animals per group per time point; 15 sections per animal were examined.

Initial observations revealed a noticeable reduction in the number of Purkinje neurons in P15 mutant rats compared to their control counterparts. In wild-type rats, Purkinje cells exhibited normal morphology, characterized by large, round nuclei (Fig. 2). The cell bodies were neatly aligned in a single layer within the Purkinje cell layer of the cerebellum. The dendritic arborizations were also visible, extending into the molecular layer. In contrast, Purkinje cells in the mutant sHW rats displayed signs of degeneration (Fig. 2). There was a reduction in the number of Purkinje cells, with many neurons appearing shrunken and fragmented, and their characteristic single-layer alignment was disrupted (Fig. 2). These findings extend previous reports, which first identified Purkinje cell abnormalities at P30, by demonstrating that degeneration is already detectable at P15. Quantitative analysis confirmed this reduction, with Purkinje cell loss ranging from 10.4% at P30 to 64.1% at P60, including a 24.6% decrease at P15 (Fig. 5a). Importantly, all quantified timepoints showed statistically significant reductions, indicating that degeneration is already measurable at P15 and intensifies across development.

**Fig. 3:**
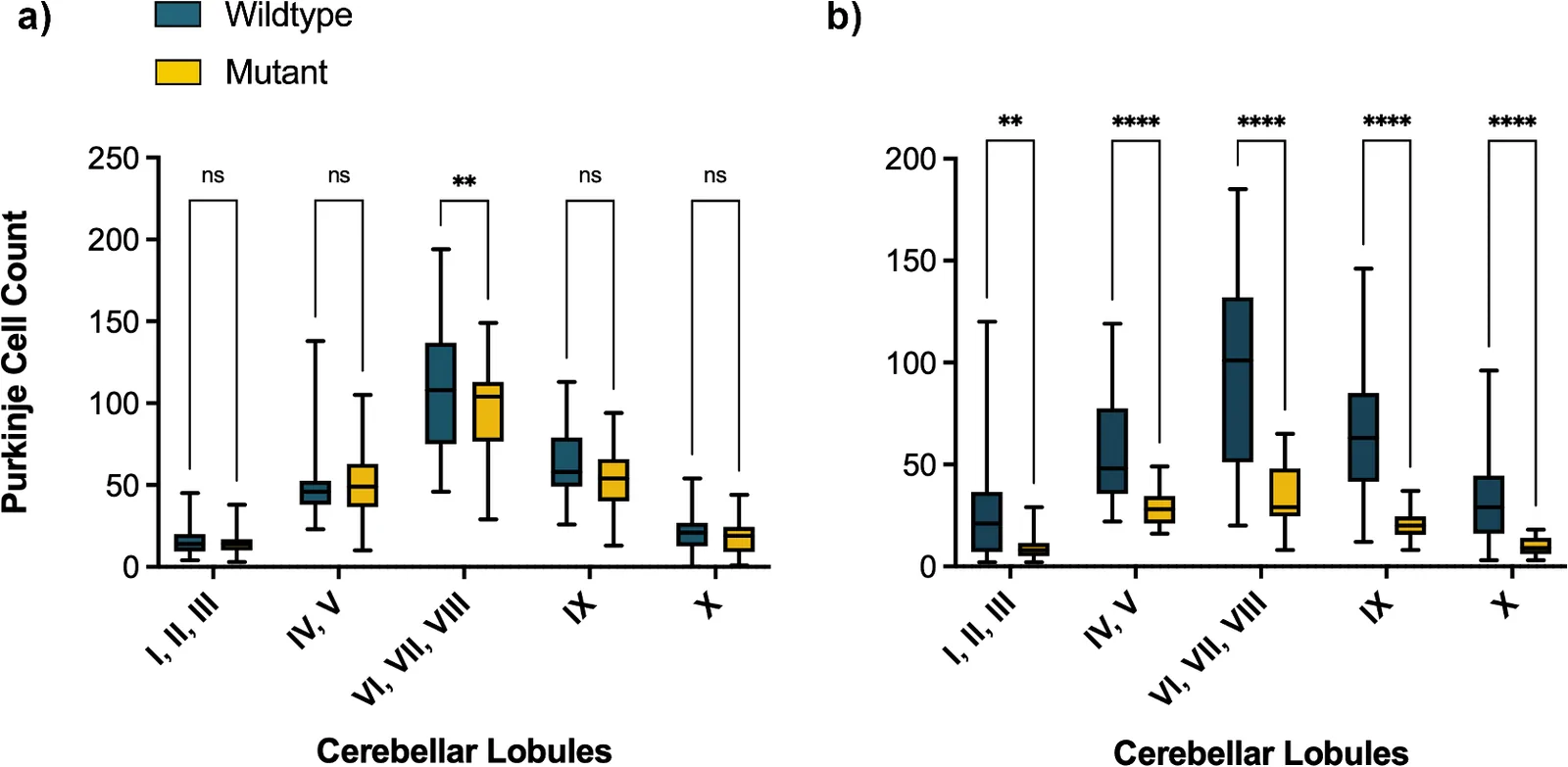
Regional per-folia Purkinje cell counts in mutant and wild-type rats at P30 and P60. (**a**) At P30, significant Purkinje cell loss was observed exclusively in the central lobules (VI-VIII). (**b**) At P60, significant reductions were observed across all lobule groups. Data represent means ± SEM (*n* = 3 animals per group; 15 sections per animal). Statistical analysis was performed using two-way ANOVA. **p < 0.001, ***p < 0.0001,* ns = not significant.*

To characterize the regional distribution of Purkinje cell loss, per-folia counts were performed at P30 and P60 across five lobule groups (Fig. 3). Per-folia analysis was not performed at P15 due to the incomplete development of cerebellar folia at this age, making lobule boundaries difficult to distinguish reliably. At P30, significant Purkinje cell loss was observed specifically in the central lobules (VI, VII, VIII) (*p* < 0.001), while other lobule groups showed no significant difference between mutant and wild-type animals. By P60, Purkinje cell loss had become widespread, with significant reductions observed across all lobule groups (lobules I-III: *p* < 0.001; lobules IV-V, VI-VIII, IX, and X: *p* < 0.0001), suggesting a progressive regional spread of degeneration throughout the cerebellum. These findings indicate that Purkinje cell degeneration in the sHW rat follows a spatiotemporal pattern, initially affecting the central lobules before spreading to encompass the entire cerebellar cortex.

### Calbindin staining confirms Purkinje cell degeneration

**Fig. 4:**
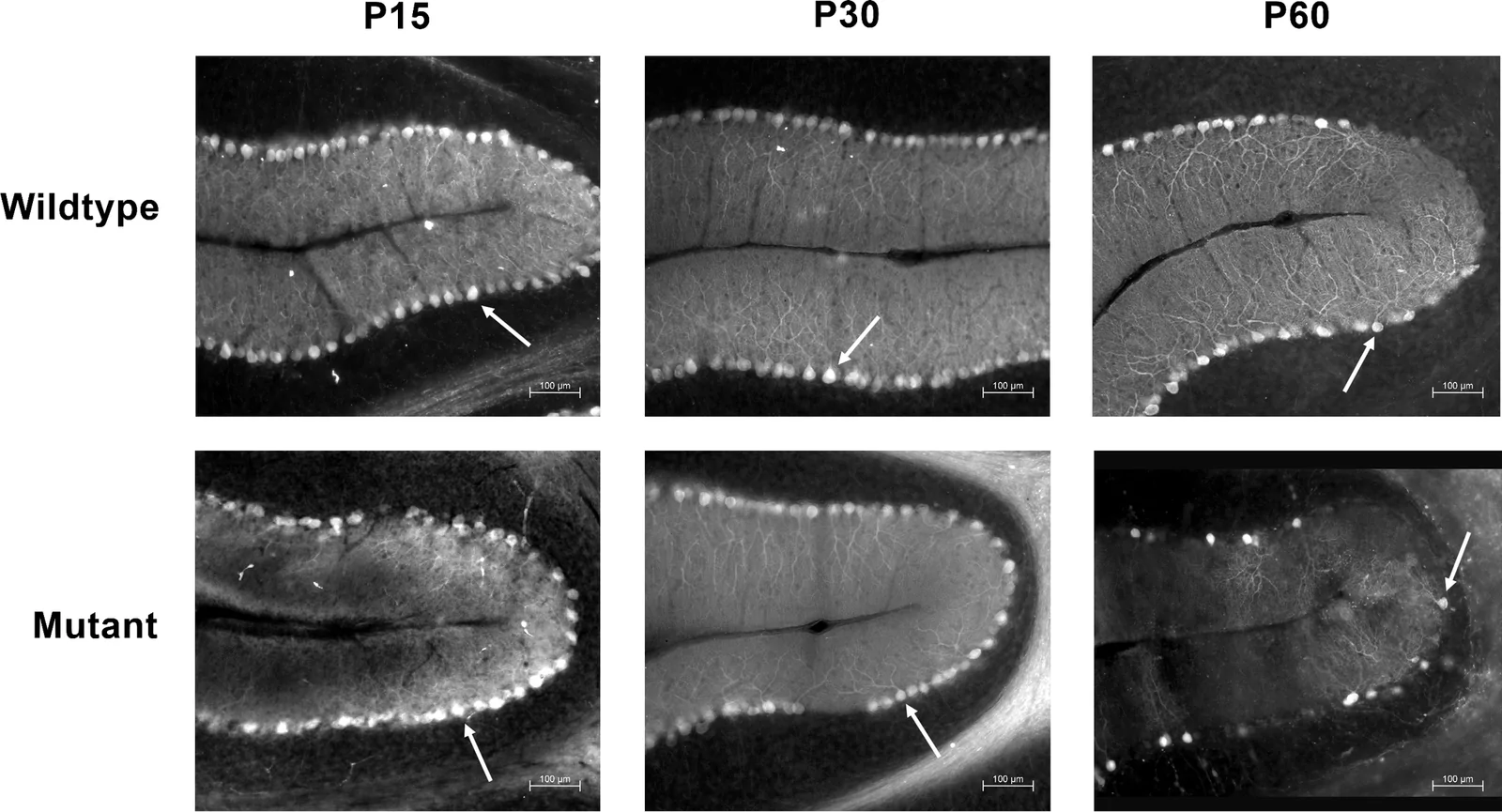
Immunohistochemical analysis of Purkinje cells in Spastic Han-Wistar rats. Representative cerebellar sections (10x; scale bar = 100 μm) from wild-type and mutant rats at P15, P30, and P60. Images are representative of the overall pattern observed throughout the cerebellum across all lobules. Purkinje cells were labeled with anti-Calbindin D-28 K antibody. Arrows indicate Purkinje cell bodies. *n* = 3 animals per group per time point; 3 sections per animal were examined.

**Fig. 5:**
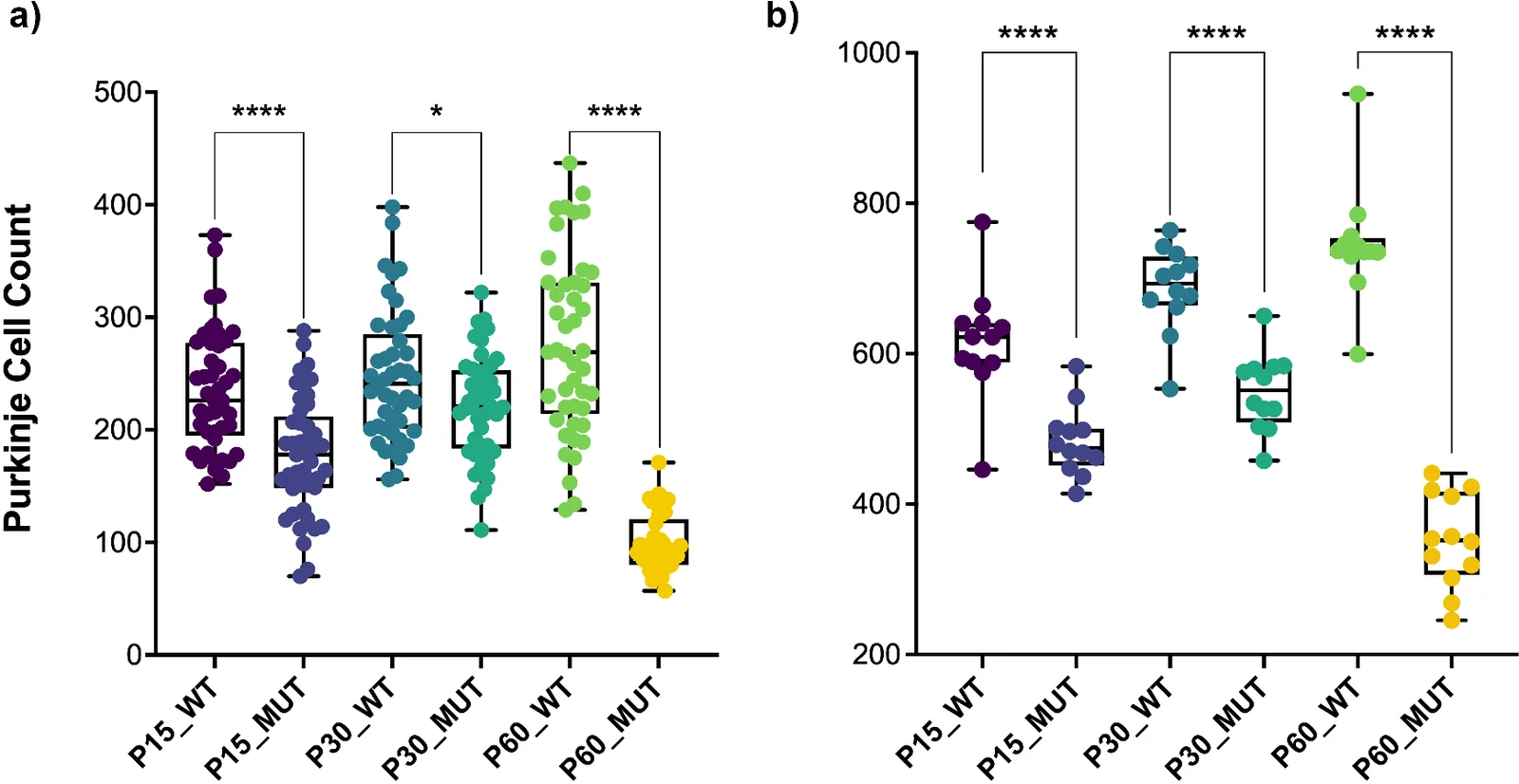
Histological and immunohistochemical quantification of Purkinje cell degeneration. (**a**) Hematoxylin & Eosin staining quantification of Purkinje cell counts at P15, P30, and P60. (*n* = 3 animals per group per time point; 15 sections per animal). Mutant rats showed a significant reduction in Purkinje cells compared to wild-type controls. (**b**) Immunohistochemical quantification of Purkinje cell counts at P15, P30, and P60. (*n* = 3 animals per group per time point; 3 sections per animal). Mutant rats showed a significantly lower number of Purkinje cells compared to wild-type controls. Data represent means ± SEM. Statistical analysis was performed using Welch’s t-test.

Calbindin immunostaining was used to provide a cell-type specific assessment of Purkinje neurons, offering greater specificity than H&E staining. Purkinje cells were visualized using the Calbindin marker in cerebellar sections from wild-type and mutant rats at P15, P30, and P60 (Fig. 4). Cerebellar sections from *n* = 3 mutant and *n* = 3 wild-type rats per time point were analyzed, with three sections per animal examined and counts averaged across sections. In wild-type animals, Purkinje cells formed a continuous monolayer with large, well-defined cell bodies and extensive dendritic arborization at all ages examined. Mutant animals had a significantly lower Purkinje cell count than age-matched wild-type controls at all time points, with reductions of 21.5% at P15, 20.0% at P30, and 52.8% at P60. Although the Purkinje cell count in mutants showed an increase from P15 to P30, it remained significantly less compared to wild-type and dropped further by P60 (Fig. 5b). These results parallel the neuronal loss observed in H&E-stained sections and provide cell-type-specific confirmation of Purkinje cell degeneration in the mutant cerebellum. To further confirm Purkinje cell dendritic degeneration, Golgi staining was performed on cerebellar sections from mutant and wild-type rats at P18 and P60. The findings corroborated the earlier onset of Purkinje cell dendritic reduction observed with Calbindin, with mutant rats displaying visibly diminished dendritic arborization compared to wild-type controls at both time points (Supplementary Fig. 1).

### Genomic search for candidate genes underlying ataxia in Han-Wistar rats

Guided by a previous pedigree analysis indicating autosomal recessive inheritance of this ataxia^19^, we used whole-genome shotgun sequencing (WGS) to search for candidate genes underlying this trait. WGS was performed on a trio from Family 1, consisting of the carrier mother, carrier father, and one affected offspring (Supplementary Fig. 2). Genome-wide average depth-of-coverage ranged from ~ 23x to ~ 25x. Family 1 included three additional siblings, one affected based on phenotypic presentation, and two asymptomatic individuals whose carrier status was unknown at the time of sample collection. These additional siblings were later used to validate candidate genes through Sanger sequencing.

Following single-nucleotide variant (SNV) calling, we searched the data for variants consistent with an autosomal recessive inheritance pattern, as previously described for this trait^17^. In doing so, we assumed the trait is not caused by a compound heterozygous mutation, which is a common cause of autosomal recessive ataxia^20^. Functional annotation classified the identified autosomal recessive variants into 17 high-impact candidates, 233 moderate-impact, and 24 low-impact, distributed across 273 genes (Supplementary Table 2). To refine this list, candidate prioritization was performed through a systematic review of publicly available resources, including peer-reviewed literature, the NCBI Gene database, the UCSC Genome Browser, and the Human Protein Atlas, to assess gene function, previously reported phenotypes, and tissue-specific expression profiles. This process identified ten genes of interest (Supplementary Table 1), one high-impact gene (*Sbf2*), six moderate-impact genes (*Rhot2*,* Sh3bp4*,* Abi2*,* Cplane1*,* Stradb*,* Eif2ak2*), and three low-impact genes (*Pkd1l2*,* Ranbp9*,* Caprin2*). We note that this is not an exhaustive list, and that the candidate genes provided (Supplementary Table 2) may serve as a resource for identifying additional potential causal variants as new functional and genomic information becomes available.

Based on predicted functional impact and biological relevance, the high-impact SNV candidate (chr1:175154510) in the *Sbf2* gene was selected for validation in Family 1 using Sanger sequencing. The segregation pattern was consistent with an autosomal recessive model: both parents were heterozygous for the variant; both phenotypically affected offspring were homozygous for the alternate allele; and the two asymptomatic siblings were confirmed to be one heterozygous carrier and one homozygous for the wildtype reference genotype. To assess whether this variant was conserved across pedigrees, *Sbf2* was subsequently evaluated in an independent Family 2 (Supplementary Fig. 2). In this case, genotypes did not segregate with the disease phenotype, and the gene was thus excluded as the causal variant.

In parallel, copy number variant (CNV) analysis was performed on the WGS data. The frataxin (*Fxn*) gene emerged as a notable candidate due to its well-documented role in hereditary ataxia and extensive prior study. *Fxn* was therefore selected for validation in Family 1; however, Sanger sequencing results were inconclusive, and no definitive segregation pattern could be established.

## Discussion

The spastic Han-Wistar rat is a well-established genetic model of hereditary ataxia, caused by a naturally inherited mutation that preferentially affects Purkinje cells in the cerebellum. Previous studies suggested that Purkinje cell degeneration emerges around P30, coinciding with the onset of observable motor impairment^18,20^. Our study extends previous work by demonstrating that Purkinje cell loss begins as early as P15, several weeks before the onset of behavioral symptoms. Such early vulnerability is consistent with observations in other ataxic mutants, such as Lurcher and *pcd* mice, where Purkinje cell loss occurs within the first postnatal weeks^22–24^. By refining the temporal onset in sHW rats, our findings address the long-standing question of when cerebellar pathology truly begins relative to symptom onset and provide a more precise disease timeline to guide biomarker discovery and pre-symptomatic intervention strategies.

Behavioral assessments in the sHW rat revealed progressive impairments in locomotion, coordination, and weight gain, with deficits becoming evident around P45. Importantly, these functional changes appeared weeks after histological evidence of Purkinje cell loss, indicating that substantial degeneration can occur before overt symptoms arise. This temporal dissociation has also been documented in spinocerebellar ataxias, where carriers often remain clinically silent despite ongoing neuronal loss^25–27^. A similar threshold-dependent effect is well established in other neurodegenerative disorders, such as Huntington’s disease, where compensatory networks delay clinical onset until a critical proportion of neurons is lost^28^. By extending this principle to the sHW rat, our findings underscore its utility in modeling the pre-symptomatic phase of hereditary ataxia and in identifying biomarkers that could inform early intervention strategies. Regional per-folia analysis further revealed that Purkinje cell loss at P30 was initially restricted to the central lobules (spinocerebellum VI-VIII), before becoming widespread across all lobule groups by P60, suggesting a widespread spatiotemporal pattern of progressive cerebellar degeneration in the sHW rat. The initial restriction of Purkinje cell loss to the central lobules is consistent with the motor coordination deficits observed in behavioral assessments, as these lobules are primarily associated with fine motor control and motor learning. This regional progression may reflect the differential vulnerability of cerebellar lobules to neurodegeneration, a pattern consistent with observations in other ataxic models and one that warrants further investigation in future studies. Golgi staining at P18 and P60 qualitatively confirmed the progressive reduction of Purkinje cell dendritic arborization in mutant animals compared to wild-type controls, corroborating the findings observed with Calbindin immunostaining (Supplementary Fig. 1).

The unresolved genetics of the sHW mutation further underscores the heterogeneity of hereditary ataxias. Our whole-genome sequencing analyses initially highlighted *Sbf2* – the gene associated with Charcot-Marie-Tooth disease in humans – as a strong candidate, with segregation patterns consistent in one pedigree. However, follow-up testing in an independent family did not support this association, leading to its exclusion as a causal variant. Similarly, copy number variation analysis suggested a possible role for *Fxn*, a gene implicated in Friedreich’s ataxia in humans, but validation results were inconclusive. Notably, recent discoveries such as the identification of pathogenic GAA repeat expansions in the *FGF14* gene, associated with SCA27B ataxia in humans, demonstrate that NGS approaches, based on short sequencing reads, may fail to detect repeat expansion disorders, underscoring an additional challenge in resolving undiagnosed cases^29^. We note that the genes listed in Supplementary Table 2 were defined as candidates based on segregation patterns in Family 1 and predicted functional impact, and have not necessarily been previously implicated in cerebellar dysfunction or related phenotypes.

Together, these findings highlight the genetic complexity of hereditary ataxia and support the clinical observation that nearly 75% of patients remain without a molecular diagnosis, despite advances in sequencing^12,13,30^. Rather than representing a limitation, this process of iterative exclusion narrows the pool of candidates and mirrors the diagnostic pathway in human patients, where excluding false leads is often as critical as identifying the causative mutation^31^. By refining the genetic search space, our work provides a valuable resource for future studies with larger cohorts and more comprehensive sequencing approaches to pinpoint the underlying variant.

The identification of early Purkinje cell degeneration in the sHW rat also has important implications beyond genetics. Establishing a cellular timeline that precedes behavioral onset provides a foundation for identifying early biomarkers of disease progression. Such markers are crucial for designing interventions during the pre-symptomatic stage, when treatments are most likely to be effective. Moreover, clarifying the onset of degeneration aligns the sHW rat more closely with human ataxias, where cerebellar atrophy is detectable years before motor symptoms appear^25,27^.

Several limitations of this study should be acknowledged. The time points examined (P15, P30, and P60) were constrained by the model’s biology, as mutant animals cannot be reliably identified before P15 and do not survive beyond P60. Histological analyses were based on a limited sample size (*n* = 3 per group per time point), and future studies with larger cohorts would strengthen these conclusions. Behavioral assessments relied on general locomotor tests; more specialized cerebellar assays may capture nuanced deficits not detected here. Finally, earlier time points could not be examined with the current phenotypic identification approach, and future molecular genotyping strategies, once the causal variant is identified, could enable characterization of earlier stages of degeneration. Regarding the genomic analyses, the following limitations apply. First, our filtering strategy assumed a simple autosomal recessive model in which the causal variant is homozygous in affected individuals. While this assumption is reasonable, we cannot discard the possibility that this trait is caused by compound heterozygous mutations. Second, our analysis focused primarily on variants within annotated genic regions. However, pathogenic variants underlying Mendelian traits are also known to occur in non-coding regulatory regions^32^, which were not systematically explored in this study. Third, the use of short-read sequencing limits the detection of certain variant types, particularly repeat expansions and structural variants in repetitive regions of the genome.

## Conclusion

In conclusion, our study demonstrates that Purkinje cell degeneration in the sHW rat begins earlier than previously recognized, preceding the onset of motor deficits by several weeks. Behavioral decline closely follows cellular loss, reinforcing the central role of Purkinje cells in ataxic pathology. Regional per-folia analysis revealed a unique spatiotemporal pattern of degeneration, with loss initially restricted to the central lobules at P30 before becoming widespread across all lobule groups by P60. In parallel, our genetic analyses excluded two high-priority candidate genes. We also present a curated list of 273 genes harboring mutations consistent with autosomal recessive inheritance, offering a valuable resource for ongoing efforts to identify the causal variant. While the complete genetic basis of the spastic Han–Wistar mutation remains to be determined, our work significantly narrows the search by ruling out unlikely candidates, providing whole-genome sequencing data for community use, and identifying promising targets for future investigation. Together, these findings establish a detailed temporal framework for cerebellar degeneration in this model and highlight its utility for investigating the early stages of hereditary ataxia and for testing pre-symptomatic therapeutic strategies.

## Methods

### Animals

Spastic Han-Wistar rats were obtained from the California State University, Northridge (CSUN) Vivarium breeding colony between the years 2023 and 2024. Both male and female rats were used, as no sex differences in phenotype have been reported in this model^19^. Animals were housed in groups of 2–4 and provided unrestricted access to standard rat chow and water. Housing was maintained on a 12 h light–dark cycle at 23–25 °C. All procedures were conducted in accordance with CSUN’s Institutional Animal Care and Use Committee (IACUC) guidelines.

### Breeding mutants

Upon weaning, animals were screened for symptoms of the mutant phenotype at approximately P15. Mutants were confirmed based on observable traits such as hyperactivity and severe whole-body tremors^17,19^. Additionally, mutants tended to exhibit decreased body weight and weaker grip strength compared to their wild-type siblings.

### Behavioral analysis

The behavioral component of this study aimed to evaluate motor coordination and activity levels in 12 mutant and 12 wild-type rats at various developmental stages. Behavioral testing was conducted by experimenters blinded to genotype. However, full blinding could not be maintained at later time points when mutant animals displayed overt motor symptoms, including severe tremors and progressive weight loss. Animals aged 9 days old were selected for assessment. Firstly, they underwent the rotarod test. Each trial was conducted at 10 RPM for a maximum of 180 s and repeated three times per subject. Subsequently, the rats were placed in a 100 × 100 cm box made of black acrylonitrile butadiene styrene (ABS) plastic. This test consisted of placing the rodent in the described box three times, each time for 120 s, during which they were free to move about and explore their environment. The animals were given a 120-second break between each run. All actions within this chamber were captured with a Logitech Quick-Cam Pro 90,000 (ffdshow codec 1:122 compression ratio, 15 fps) andrecorded to a computer hard drive using VirtualDub. Video tracking and movement analysis were conducted using custom scripts written in Python with the OpenCV computer vision library (opencv.willowgarage.com). Lastly, the weight of each animal was recorded to monitor potential correlations between behavioral performance and physical development. These three tests were conducted every 5 days, starting when the rats were 9 days old and continuing until they reached 65 days of age.

### Brain fixation and harvesting

At the designated age, the animals were euthanized, and the brains were extracted for analysis. This process involved exposing the animals to CO_2_ followed by a transcardiac perfusion. Animals were placed in a perfusing pan, and a midline incision through the sternum was made to expose the heart for upper body perfusion using transcardiac puncture. Using hemostats, the descending aorta was clamped to prevent whole-body perfusion. A 26-gauge needle was inserted into the left ventricle, the right atrium was then immediately incised, and a peristaltic pump was activated with a flow rate of 7 ml per minute. Animals were perfused with a 0.9% saline solution to clear most of the blood from the head and neck region. The paleness of the eyes, ears, and forelimbs visually confirmed clearance of blood, and the stiffness of the neck further confirmed clearance of blood. Afterward, 4% paraformaldehyde at pH 7.4 was used on the animal tissue until complete fixation was achieved. The perfusion is also confirmed by physical inspection of increased stiffness in the neck. The brain was then removed, placed in 4% paraformaldehyde, and stored at 4 °C for future use.

### Tissue sectioning and mounting by cryostat

Cerebellums of harvested brains were sectioned using a cryostat microtome, Leica CM1950, and mounted on a cryostat-mounting platform using tissue freezing media (Tissue-Tek OCT compound 4583). These brains were then sectioned at -20 °C into 15 μm sagittal cuts. After sectioning, the sections were promptly mounted onto superfrost slides and allowed to dry for 24–48 h in a 4 °C refrigerator to ensure proper adhesion before staining. For each animal, fifteen randomly selected tissue sections were used for H&E staining.

### Hematoxylin and eosin staining

The morphology and structural integrity of Purkinje cells were evaluated using Harris’s Hematoxylin and Eosin staining. Cerebellar sections from mutant rats and wild-type littermates were processed in parallel. Sections were rehydrated through graded ethanol (100% and 95%), immersed in hematoxylin for five dips (with staining duration adjusted to achieve optimal intensity), and rinsed in tap water. Slides were differentiated in 1% acid alcohol (1 mL HCl in 99 mL 95% ethanol), rinsed in 2% aqueous sodium bicarbonate to enhance nuclear staining, and counterstained with eosin Y for 20 s. Following dehydration in graded ethanol, slides were cleared in xylene and coverslipped using Permount. Purkinje cell morphology and evidence of neuronal degeneration were assessed in mutants at postnatal days 15, 30, and 60 using light microscopy. Sagittal sections were sampled throughout the cerebellum, spanning all lobules. Purkinje cells were counted manually per folia across all cerebellar lobules, and counts were averaged across sections for each animal. For regional analysis, lobules were grouped into five regions corresponding to the five classic cardinal lobes of the mammalian cerebellum: anterior basal lobe (lobules I-III), anterior dorsal lobe (lobules IV-V), central lobe (lobules VI-VIII), posterior lobe (lobule IX), and inferior lobe (lobule X)^33^. Regional per-folia analysis was performed at P30 and P60 only, as cerebellar folia at P15 are not yet fully developed, making individual lobule boundaries difficult to distinguish reliably. Cell counting was performed by lab members blinded to genotype where possible; however, partial blinding was maintained as the experimenter responsible for animal handling and tissue processing was aware of genotype assignments.

### Tissue embedding and vibratome sectioning

For IHC, cerebella were embedded in 4% agarose prepared in 1× phosphate-buffered saline (PBS). To prepare the agarose, 4 g of agarose powder was added to 100 mL 1× PBS, allowed to hydrate at room temperature, and then heated in a microwave oven for ~ 2 min with intermittent swirling every 30 s to prevent boiling and foaming, until fully dissolved. The solution was cooled to approximately 40 °C before embedding to avoid heat damage to the tissue. Tissues were placed in appropriately sized disposable plastic molds, and molten agarose was gently poured to fully submerge the tissue, ensuring it was lifted slightly so that a thin agarose layer separated the tissue from the mold base. Once the agarose had solidified at room temperature, blocks were wrapped in a damp paper towel, sealed in plastic bags, and stored at 4 °C for up to 24 h before sectioning.

For vibratome sectioning, excess agarose surrounding the tissue was trimmed, and blocks were mounted with the desired orientation maintained. Sagittal sections of cerebellum were cut at 100 μm thickness using a vibratome (Leica, VT1000S) according to the manufacturer’s instructions. Sections for IHC were collected into wells containing 4% paraformaldehyde (PFA) for storage before staining.

### Immunohistochemistry

Free-floating cerebellar sections were processed for IHC staining to visualize Purkinje neurons. Sections were transferred into new wells and blocked with 10% goat serum in phosphate-buffered saline (PBS) for 4 h at 37 °C to prevent nonspecific binding. Following blocking, the tissues were rinsed twice with 1× PBS and then incubated in 600 µL PBS containing the anti-Calbindin D-28 K primary antibody (4 µL per well), a marker for Purkinje cells (*1:500*,* Abcam*,* Cat# ab108404)*. Sections were incubated for 48 h at 37 °C. After primary antibody incubation, sections were washed six times with PBS (30 min each) on a gentle shaker, adding PBS along the side of the well to minimize tissue disruption. Samples were then incubated overnight at 37 °C with Alexa Fluor 488 conjugated anti-rabbit (4 µL/well) secondary antibodies. The following day, sections were washed six times with PBS (30 min each) using the same gentle washing method. Sections were mounted on slides, coverslipped, and imaged using a Zeiss fluorescence microscope equipped with appropriate filter sets for Alexa Fluor 488 (*1:500*,* Invitrogen*,* Cat# A-11008)*. Images were acquired at consistent exposure times, saved as high-resolution digital files, and processed using AxioImage software (Zeiss). Sagittal sections were sampled throughout the cerebellum, spanning all lobules. Purkinje cells were counted manually as total counts per section, with three sections analyzed per animal, and counts were averaged across sections for each animal. Cell counting was performed by lab members blinded to genotype where possible; however, partial blinding was maintained as the experimenter responsible for animal handling and tissue processing was aware of genotype assignments.

### Golgi staining

Purkinje cell dendritic morphology was assessed using the FD Rapid Golgi-Stain™ Kit protocol (FD Neuro-Technologies, PK401). Freshly dissected cerebellar tissue from mutant and wild-type rats at P18 and P60 (*n* = 3 per group) was rinsed briefly in double-distilled water to remove surface blood. Tissue was then immersed in an impregnation solution prepared by mixing equal volumes of Solutions A and B at least 24 h prior to use and stored at room temperature for 2 weeks in the dark. The impregnation solution was replaced after the first 6 h of immersion. Following impregnation, tissue was transferred to Solution C and stored at 4 °C for at least 48 h in the dark, with the solution replaced after the first 24 h. The tissue was sectioned at 100 μm using a vibratome (Leica, VT1000S). Each section was mounted on gelatin-coated microscope slides using Solution C. Excess solution was absorbed with filter paper, and sections were allowed to dry naturally at room temperature. Dried sections were rinsed twice in double-distilled water, placed in a working solution consisting of 1 part Solution D, 1 part Solution E, and 2 parts double-distilled water for 10 min, and rinsed again twice in double-distilled water. Sections were then dehydrated through graded ethanol (50%, 75%, 95%, and 100%), cleared in xylene, and coverslipped using Permount. Purkinje cell dendritic arborization was examined and imaged using light microscopy.

### Genetic mapping

A family trio was selected for whole-genome sequencing (WGS), comprising carrier parents, approximately one year of age, and an affected offspring. Both parents were heterozygous carriers of the spastic Han-Wistar allele, as they had previously produced progeny with and without the disease phenotype^19^. In the litter from which the trio was derived, an additional two affected and two unaffected offspring were identified at 60 days of age for further DNA extraction and Sanger confirmation of potential candidate genes. Pedigrees are shown in Supplementary Fig. 1. Total DNA was extracted from liver tissue samples obtained from mutants and wild-type rats using the DNeasy Blood & Tissue Kit (Qiagen, Germany), following the manufacturer’s protocol.

Raw sequencing reads were processed using the Illumina DRAGEN Germline Pipeline (v4.3.13), aligned to the *Rattus norvegicus* reference genome (Rnor_6.0). The DRAGEN pipeline performs key steps, including read mapping, sorting, duplicate marking, and germline variant calling. In each step, the default parameters from the DRAGEN pipeline were used. Quality control metrics were summarized using MultiQC. Variant Call Format (VCF) files generated from Germline analyses were annotated using the Ensembl Variant Effect Predictor (VEP, v113) with the Ensembl RN6.104 annotation set. VEP was used to determine the predicted functional consequences of each variant. CNV calling was performed using the previously generated BAM files and the DRAGEN Germline pipeline (v4.3.13). Segmentation was conducted using the Shifting Level Model.

Candidate gene analysis was performed by filtering both annotated SNV and CNV datasets to identify variants following an autosomal recessive inheritance pattern. For SNVs, variants where the father and mother exhibited heterozygous genotypes (0/1) while the proband displayed homozygous alternate genotypes (1/1) were extracted. For CNVs, only deletions following the same genotype pattern were considered, with additional copy number requirements of copy number = 1 in both parents and copy number = 0 in the proband. Functional impact prioritization was performed using VEP impact predictions, with variants classified as HIGH impact (transcript ablation, stop gained, stop loss, frameshift variants) and MODERATE impact (missense variants, in-frame indels) prioritized for downstream analysis. The resulting candidate gene list represented loci harboring putative pathogenic recessive variants that could explain the observed ataxia phenotype in the proband. All filtering and analysis steps were performed using bedtools^34^ and custom shell scripts.

### Candidate gene validation

Primers were designed for Polymerase Chain Reaction (PCR) experiments based on candidate genes identified from the WGS results using Primer3 software (https://primer3.ut.ee/*).* PCR reactions were performed using 100 ng of genomic DNA in a reaction mixture containing 1× buffer, 4 mM MgCl₂, 0.2 mM dNTPs, 0.4 µM forward and reverse primers, and 0.05 U/µL Taq DNA polymerase. Thermal cycling conditions were optimized as follows: an initial denaturation at 95°C for 2 min; 30 cycles of denaturation at 95°C for 30 s, annealing at 60°C for 30 s, and extension at 72°C for 1 min; and a final extension at 72°C for 7 min. The primers used were as follows: *Sbf2* forward: 5′- ACATCTCAGCACCCGCTTCC-3′, reverse: 5′-TCACAGTAGCACGGAATTTGA-3′; *Fxn* forward: 5′-ATCTGATGCCCCTCTTCTGG-3′, reverse: 5′-TGGGAGTGAATTCAGGGCTT − 3’.

PCR products were purified using the ExoSAP-IT Express kit (Thermo Scientific, USA) according to the manufacturer’s protocol. Sequencing reactions were prepared in a final volume of 10 µL using the BigDye Terminator v3.1 Cycle Sequencing Kit (Thermo Scientific, USA), with 1 µL of purified PCR product and 1 µM of either forward or reverse primer. Thermal cycling for sequencing consisted of an initial denaturation at 96 °C for 1 min, followed by 25 cycles of 96 °C for 10 s, 50 °C for 5 s, and 60 °C for 45 s, with a ramp rate of 1 °C/s. Sequencing products were purified using the BigDye XTerminator Purification Kit (Thermo Scientific, USA) and analyzed on a SeqStudio Genetic Analyzer (Thermo Scientific, USA). The resulting sequences were aligned to the *Rattus norvegicus* reference genome assembly Rnor_6.0 (rn6) using Geneious R9 software, and genotypes for the selected genes were annotated accordingly.

## Supplementary Information

Below is the link to the electronic supplementary material.

Supplementary Material 1

## Data Availability

Raw whole-genome sequencing data (FASTQ files) from the parent-offspring trio have been deposited in the NCBI Sequence Read Archive (SRA) under BioProject accession PRJNA1474998 (BioSamples: SAMN60625060, SAMN60625061, and SAMN60625062). All other datasets generated and/or analyzed during the current study are available from the corresponding author upon reasonable request.
