# Supplementary material for: Purkinje cell development and degeneration in the spastic Han-Wistar rat model of ataxia
Source: Sci Rep. 2026 Jun 25;16:29223. doi: 10.1038/s41598-026-58826-y (PMC13590475; doi:10.1038/s41598-026-58826-y)
Supplement: Supplementary file 1 [file 41598_2026_58826_MOESM1_ESM.docx]

**Supplementary**

**Supplementary Figure 1. Golgi staining of Purkinje cells in wild-type and mutant sHW rats.** Representative Golgi-stained cerebellar sections (20x and 40x) showing Purkinje cell dendritic morphology in wild-type and mutant rats at P18 and P60. Arrows indicate Purkinje cells with well-developed dendritic arborization in wild-type rats. In contrast, mutant Purkinje cells show progressively reduced and disorganized dendritic arborization, with markedly simplified branching patterns by P60.

**Supplementary Figure 2. Pedigrees for candidate gene validation**. In Family 1, whole-genome sequencing was performed on the mother, father, and one affected offspring. Sanger sequencing of the *Sbf2* gene was performed for all individuals in both families.

**Supplementary Table 1.** Candidate genes identified from WGS and filtered by predicted impact and disease relevance.

| **Gene Symbol** | **Predicted Impact** | **Chromosome Position** | **Variant** | **Predicted Function** | **Associated diseases in humans** | **Brain expression**  **(Human Atlas Protein)** |
| --- | --- | --- | --- | --- | --- | --- |
| *Sbf2** | High | chr1:175154510 | ENSRNOT00000013126.7:c. 1788+16789_1788+16792del | phosphatase binding activity | Charcot-Marie-Tooth Disease, Type 4B2 | Neuronal and Glial cells |
| *Rhot2* | Moderate | chr10:15207579 | ENSRNOT00000027083.5:c.1277G>A\|ENSRNOP00000027083.3:p.Arg426Gln | GTP binding activity and  GTPase activity | Charcot-Marie-Tooth Disease Type 4; [3-Methylglutaconic Aciduria, Type I](http://www.malacards.org/card/3_methylglutaconic_aciduria_type_iii)II | Cerebellum; Neuronal cells |
| *Sh3bp4* | Moderate | chr9:96279068 | ENSRNOT00000026312.4:c.1789G>A\|ENSRNOP00000026312.2:p.Ala597Thr | GDP-dissociation inhibitor  activity and small GTPase  binding activity | Charcot-Marie-Tooth Disease Type 1A | Glial cells |
| *Abi2* | Moderate | chr9:67282241 | ENSRNOT00000050179.5:c.1253A>G\|ENSRNOP00000050008.5:p.Tyr418Cys | SH3 domain binding activity;  proline-rich region binding activity;  and ubiquitin protein ligase  binding activity. | [Developmental And Epileptic Encephalopathy 65](http://www.malacards.org/card/developmental_and_epileptic_encephalopathy_65); Autosomal Recessive Limb-Girdle Muscular Dystrophy Type 2H | Neuronal and Glial cells |
| *Cplane1* | Moderate | chr2:57324788  chr2:57332058  chr2:57332208 | ENSRNOT00000063899.3:c.5657A>G\|ENSRNOP00000060886.3:p.Asp1886Gly  ENSRNOT00000063899.3:c.6478G>A\|ENSRNOP00000060886.3:p.Ala2160Thr  ENSRNOT00000063899.3:c.6628T>C\|ENSRNOP00000060886.3:p.Ser2210Pro | Cilium assembly | [Ciliopathies](https://pathcards.genecards.org/card/ciliopathies); Joubert syndrome | Neuronal and Glial cells |
| *Stradb* | Moderate | chr9:65781702 | ENSRNOT00000043558.2:c.1178C>T\|ENSRNOP00000049160.2:p.Ala393Val | Activation of protein kinase activity  and protein export from nucleus | Amyotrophic Lateral Sclerosis 2, Juvenile. | Neuronal and Glial cells (low) |
| *Eif2ak2* | Moderate | chr6:1431196 | ENSRNOT00000072810.2:c.1387G>A\|ENSRNOP00000067694.2:p.Val463Ile | Double-stranded RNA binding activity; eukaryotic translation initiation factor 2alpha kinase activity | [Dystonia 33](http://www.malacards.org/card/dystonia_33); Leukoencephalopathy, Developmental Delay; Episodic Neurologic Regression Syndrome | Neuronal and Glial cells |
| *Pkd1l2* | Moderate | chr19:49550350  chr19:49592225  chr19:49596537 | ENSRNOT00000071130.2:c.6196G>A\|ENSRNOP00000064630.2:p.Asp2066Asn  ENSRNOT00000071130.2:c.2488C>T\|ENSRNOP00000064630.2:p.Arg830Cys  ENSRNOT00000071130.2:c.2332G>A\|ENSRNOP00000064630.2:p.Val778Met | Calcium channel activity; detection  of mechanical stimulus | Otosclerosis 4; [Long Qt Syndrome](http://www.malacards.org/card/long_qt_syndrome) | Neuronal and Glial cells |
| *Randp9* | Moderate | chr17:24021748  chr17:24021760 | XM_008771586.1:c.2246G>A\|XP_008769808.1:p.Arg749Lys  XM_008771586.1:c.2234C>T\|XP_008769808.1:p.Ser745Leu | Cytoskeleton organization and negative regulation of ERK1 and ERK2 cascade | [Masa Syndrome](http://www.malacards.org/card/masa_syndrome); Lissencephaly | Neuronal and Glial cells |
| *Caprin2* | Moderate | chr4:183346989 | ENSRNOT00000074740.3:c.849+1573A>G | Positive regulation of canonical Wnt signaling pathway; positive regulation of dendritic spine morphogenesis; positive regulation of transcription by RNA polymerase II | Combined Oxidative Phosphorylation Deficiency 32 | Neuronal and Glial cells |

**Supplementary Table 2**. Functional annotation classified the variants into 273 genes (high, moderate, low).

**High Impact**

LOC498152

Olr1593

Gsr

AABR07027267.1

Cep192

AABR07043288.1

AABR07003433.1

Sbf2

AABR07005821.1

Slc22a12

RGD1624210

Bche

AABR07007839.1

Eef1akmt3

AABR07057436.1

Snrpe

Mettl21cl1

**Moderate Impact**

Enpp7

Rhot2

Prr35

Capn15

Olr1401

Abca9

Tmprss15

AABR07033357.1

Htr1f

AABR07033047.1

Olr1539

Kng1

LOC498152

LOC102546864

LOC498154

Adap1

Zfp68

MGC112692

LOC288521

Cyp3a9

Mcm7

Taf6

Mblac1

Vom2r62

Vom2r64

AABR07035776.1

Ache

AABR07035791.1

Atp6v0a2

Mphosph9

AABR07035074.1

Vom2r60

Rps6kc1

Flvcr1

Nsl1

AABR07022157.1

Hhat

Sertad4

Traf3ip3

Hsd11b1

G0s2

Lamb3

Cntnap5b

AABR07021804.1

Mnda

Olr1585

Olr1587

Olr1592

Olr1593

Olr1595

Olr1596

Spta1

Olr1600

AABR07021812.1

AABR07021930.1

LOC498300

Nvl

Ccdc121

RGD1565660

Vom2r73

Evc2

Evc

Wfs1

Olr1611

Olr1297

AABR07026048.1

AABR07024441.1

Kcnu1

Defa8

AABR07026536.1

F10

AABR07027235.1

Prl7a3

Prl7b1

Prl8a7

AABR07027637.1

Prtfdc1

Thnsl1

AABR07028839.1

AABR07043288.1

Pkd1l2

Hsd17b2

Htatip2

AABR07003433.1

Ulbp1

Agbl1

Thap12

Stk33

Trim66

Akip1

LOC499240

Lyve1

Bub3

Slc18b1

Trip13

Vom1r6

LOC102552527

LOC102555377

Zfp579

Sult2a1

Rpl9

Bicra

Zfp541

LOC108349752

Psgb1

AABR07002659.1

LOC102557244

AC093995.2

Mill1

Ppp1r13l

AABR07002680.2

Ceacam19

Ceacam20

AABR07002683.1

Zfp111

Zfp61

C4a

AABR07044383.1

LOC103690108

RGD1624210

RT1-CE4

AABR07044408.1

AABR07010333.1

Vom2r46

AABR07011057.1

Wdr49

Abca4

Dnttip2

Pde5a

Synpo2

Prss12

Enpep

Tbck

Cxxc4

Slc9b2

Manba

Odf2l

Nexn

Ak5

Arhgef28

Gfm2

Hexb

Tmem174

Map1b

Marveld2

Cdk7

Cenph

Cplane1

Pdzd2

Slc10a5

AC121203.3

Slc28a2

LOC680432

Ckap2l

Il1a

Aar2

LOC100359951

Lypd6b

Erich2

Slc25a12

Cerkl

Olr528

Olr701

Fgl2

AABR07059243.1

Mthfd2

Cd207

Magi1

Ier3ip1

AABR07061532.1

AABR07061614.1

Il17rc

Olr829

Plekhg6

Samd9

AABR07059663.1

Galnt11

Agap3

Atg9b

Nupl2

Doxl2

Orc5

Lypla1

Fbxo10

Frmpd1

AABR07048419.2

AABR07065139.2

Msh6

AC123293.2

Eif2ak2

Cebpz

Ndufaf7

Klf11

LOC108349290

Vezt

Fgd6

AABR07056686.1

Olr956

Tspan31

RGD1565117

Nxph4

LOC100365958

Tac3

Gpr182

AABR07057436.1

Stag1

Col6a6

Col6a5

Susd5

Tcaim

Zkscan7

RGD1311745

Htr3b

Dus3l

AABR07066648.1

Satb1

Smap1

AABR07067520.1

Col5a2

Asnsd1

RGD1306941

Sgo2

Aox1

Aox3

Aox4

Stradb

AABR07067814.1

Abi2

Vom2r79

Adgre1

Sh3bp4

C3

Dennd1c

**Low Impact**

Cadm2

Adap1

Cyp3a62

AABR07035074.1

Spta1

Gsr

Ranbp9

Pkd1l2

Plcg2

Zfp61

LOC103690108

Hexb

Il1a

Gpd2

Hoxd9

Caprin2

AABR07059663.1

Fbxo10

Mon2

AABR07057436.1

Cnot10

Aox3

AABR07067810.2

Aox4
